# *CYP3A5* influences oral tacrolimus pharmacokinetics and timing of acute kidney injury following allogeneic hematopoietic stem cell transplantation

**DOI:** 10.3389/fphar.2023.1334440

**Published:** 2024-01-08

**Authors:** Nathan D. Seligson, Xunjie Zhang, Mark C. Zemanek, Jasmine A. Johnson, Zachary VanGundy, Danxin Wang, Mitch A. Phelps, Julianna Roddy, Craig C. Hofmeister, Junan Li, Ming J. Poi

**Affiliations:** ^1^ Division of Pharmacy Practice and Science, College of Pharmacy, The Ohio State University, Columbus, OH, United States; ^2^ Department of Pharmacy, The Ohio State University Wexner Medical Center, Columbus, OH, United States; ^3^ Comprehensive Cancer Center, The Ohio State University, Columbus, OH, United States; ^4^ Department of Pharmacotherapy and Translational Research, College of Pharmacy, University of Florida, Gainesville, FL, United States; ^5^ Division of Pharmaceutics and Pharmaceutical Chemistry, College of Pharmacy, The Ohio State University, Columbus, OH, United States; ^6^ Department of Hematology and Oncology, Winship Cancer Institute of Emory University, Atlanta, GA, United States; ^7^ Division of Outcomes and Translational Sciences, College of Pharmacy, The Ohio State University, Columbus, OH, United States

**Keywords:** tacrolimus, single nucleotide polymorphism, precision medicine, hematologic stem cell transplant, CYP3A4, CYP3A5

## Abstract

**Introduction:** Polymorphisms in genes responsible for the metabolism and transport of tacrolimus have been demonstrated to influence clinical outcomes for patients following allogeneic hematologic stem cell transplant (allo-HSCT). However, the clinical impact of germline polymorphisms specifically for oral formulations of tacrolimus is not fully described.

**Methods:** To investigate the clinical impact of genetic polymorphisms in *CYP3A4*, *CYP3A5*, and *ABCB1* on oral tacrolimus pharmacokinetics and clinical outcomes, we prospectively enrolled 103 adult patients receiving oral tacrolimus for the prevention of graft-versus-host disease (GVHD) following allo-HSCT. Patients were followed in the inpatient and outpatient phase of care for the first 100 days of tacrolimus therapy. Patients were genotyped for *CYP3A5* *3 (rs776746), *CYP3A4* *1B (rs2740574), *ABCB1* exon 12 (rs1128503), *ABCB1* exon 21 (rs2032582), *ABCB1* exon 26 (rs1045642).

**Results:** Expression of *CYP3A5* *1 was highly correlated with tacrolimus pharmacokinetics in the inpatient phase of care (*p* < 0.001) and throughout the entirety of the study period (*p* < 0.001). Additionally, Expression of *CYP3A5* *1 was associated with decreased risk of developing AKI as an inpatient (*p* = 0.06). Variants in *ABCB1* were not associated with tacrolimus pharmacokinetics in this study. We were unable to discern an independent effect of *CYP3A4* *1B or *22 in this population.

**Conclusion:** Expression of *CYP3A5* *1 is highly influential on the pharmacokinetics and clinical outcomes for patients receiving oral tacrolimus as GVHD prophylaxis following allo-HSCT.

## 1 Introduction

Allogeneic hematopoietic stem cell transplantation (allo-HSCT), a mainstay treatment for a variety of hematologic malignancies (Sureda et al., 2015), is associated with a significant risk of graft-versus-host disease (GVHD) (Ziakas et al., 2014). Therapeutic immune suppression, often utilizing tacrolimus, is necessary to reduce the risk of GVHD (Hiraoka et al., 2001). Significant inter-patient variability in the pharmacokinetics of tacrolimus, along with a narrow therapeutic window, makes dosing of tacrolimus difficult. Sub-therapeutic levels of tacrolimus are associated with an increased risk of GVHD, while supra-therapeutic levels are associated with severe toxicity (Prezepiorka et al., 1999; Jacobson et al., 2001; Lee et al., 2002; Musuamba et al., 2009; Levine et al., 2010). Clinically, therapeutic drug monitoring of tacrolimus has been employed to personalize tacrolimus doses for patients and account for the known inter-patient pharmacokinetic and pharmacodynamic variability of tacrolimus (Elens et al., 2007; Thervet et al., 2008; Brunet et al., 2019).

Out of many factors associated with inter-patient pharmacokinetic and pharmacodynamics variabilities of tacrolimus, single nucleotide polymorphisms (SNPs) in *cytochrome* (*CYP*) *3A4* and *3A5* enzymes have been associated with clinical outcomes associated with tacrolimus (Kuehl et al., 2001; Thervet et al., 2010; Elens et al., 2011; Wang et al., 2011). The *CYP3A5* *3 allele, the most frequently occurring *CYP3A5* allele in populations of European ancestry, references an intronic SNP rs776746 that creates a cryptic splice site and an aberrant splice isoform with partial intron insertion, resulting in a premature stop codon and eliminating the expression of the functional CYP3A5 enzyme (Kuehl et al., 2001; Thervet et al., 2010). While the *CYP3A5* *3 allele is the most prevalent allele, individuals with the wild type allele (designated *1) are considered *CYP3A5* *1 expressers (*1/*1 or *1/*3). *CYP3A5* *1 expressers required increased tacrolimus dose to maintain therapeutic levels of the drug compared to patients with the *CYP3A5* *3/*3 genotype (Thervet et al., 2010; Khaled et al., 2016; Hamadeh et al., 2019). *CYP3A4* also plays a critical role in tacrolimus metabolism (Elens et al., 2011; Wang et al., 2011). Two specific *CYP3A4* alleles have been linked to variable tacrolimus metabolism: *CYP3A4* *22, which refers to a polymorphism in intron 6 of *CYP3A4* that reduces expression of the *CYP3A4* mRNA and protein (Wang et al., 2011; Wang and Sadee, 2016), and *CYP3A4* *1B which refers to a SNP on the promoter region of *CYP3A4* whose impact remains to be further elucidated (Garcia-Martin et al., 2002; Spurdle et al., 2002; Gervasini et al., 2012; Zochowska et al., 2012). A*denosine triphosphate-binding cassette B1* (*ABCB1*) is a cell membrane transporter responsible for the active transport of tacrolimus (Op den Buijish et al., 2007). It has been proposed that *ABCB1* plays a role in the bioavailability of tacrolimus in the setting of oral administration; however, little is known about the specific impacts of *ABCB1* genomic variants on the pharmacokinetic and pharmacodynamic properties of tacrolimus in transplant patients (Mourad et al., 2006; Hawwa et al., 2009; Herrlinger et al., 2011; Kim et al., 2012; Tapiradamaz et al., 2014; Li et al., 2015; Debette-Gratien et al., 2016).

In the 2015 guidelines for the use of pharmacogenomic data to guide tacrolimus dosing, the Clinical Pharmacogenetics Implementation Consortium (CPIC) recommended that patients expressing *CYP3A5* *1 should receive doses 1.5-2-fold higher compared to standard dosing (Birdwell et al., 2015). While this recommendation is made for kidney, heart, lung, and allo-HSCT patients, it is notable that such recommendation in the setting of allo-HSCT is made based on evidence provided for intravenous tacrolimus (Woodahl et al., 2008; Yanagimachi et al., 2010; Onizuka et al., 2011; Yanagisawa et al., 2011; Iwamoto et al., 2015; Khaked et al., 2016; Hamadeh et al., 2019; Suztsugu et al., 2019). Very little data is available for patient receiving allo-HSCT alongside oral tacrolimus (Zhu et al., 2020). As such, clinicians have to extrapolate the findings of intravenous tacrolimus studies when considering this clinical problem. In addition to hepatic expression, CYPs in the 3A family are highly expressed in the intestine and are detectable in some brain regions (McFadyen et al., 1998; Paine et al., 2006). Given the potential for intestinal metabolism, as well as first-pass hepatic metabolism prior to reaching systemic circulation, the effect of genomic variants on tacrolimus administered orally poses an additional level of complexity in comparison to intravenous tacrolimus. While significant data exists demonstrating the role of pharmacogenomic variants on oral tacrolimus outside of allo-HSCT (Goto et al., 2004; Kato et al., 2018; Pasternak et al., 2019; Pasternak et al., 2022), this data may not fully apply in the setting of allo-HSCT given the differences in tacrolimus dosing and a desire for less aggressive early tacrolimus levels to confer a graft-versus-leukemia effect (Imado et al., 2004). It is therefore possible that existing findings using intravenous formulations of tacrolimus in the setting of allo-HSCT, and those of oral tacrolimus outside of allo-HSCT, may not fully reflect the pharmacogenomic implications of tacrolimus metabolism specific to the oral route in an allo-HSCT population. In this study, we present a prospective analysis of 103 patients who received oral tacrolimus for the prevention of GVHD following allo-HSCT.

## 2 Materials and methods

### 2.1 Study population

A cohort of 103 adult patients receiving oral tacrolimus for the prevention of GVHD following allo-HSCT at The Ohio State University Wexner Medical Center (OSUMC) were prospectively enrolled into this study following approval from the Cancer Institutional Research Board’s guidance (IRB# 2012C0021) from 06/2012 until 07/2014. Inclusion criteria included age great than 18 years, patient to receive their first allo-HSCT during the study period, and absence of renal or hepatic dysfunction at baseline. Exclusion criteria included HIV positivity, presence of any medical conditions that would interfere with the patient’s ability to sign the informed consent, hypersensitivity to any component of tacrolimus, clinical history of any solid organ transplantation, previous chronic use of tacrolimus, or pregnancy.

### 2.2 Study procedures

Following patient consent, but prior to transplantation, whole blood samples were obtained to determine the patient’s *CYP3A4*, *CYP3A5*, *ABCB1* genotypes. Clinical data was collected for patients for up to the first 100 days post-transplant or until the patient was lost to follow-up. As a standard of care in HSCT patients treated at OSUMC, tacrolimus therapeutic drug monitoring was employed using liquid chromatography/tandem mass spectrometry at least three times per week to measure trough drug concentration until goal steady state concentrations were reached. Standard of care at our institution includes starting immediate release oral tacrolimus 2 days prior to transplantation at a dose of 0.03 mg/kg. Twice daily dosing was considered standard; however, clinicians could elect to use daily dosing if necessary for the patient. Tacrolimus levels were normalized by both tacrolimus doses received as well as patient’s actual body weight as described in previous studies as follows: Normalized Tacrolimus level (ng/dL/mg/kg) = Tacrolimus level (ng/dL)/Total daily dose of Tacrolimus (mg)/Actual body weight (kg) (Sy et al., 2013; Cusinato et al., 2014; Ito et al., 2017).

To assess the proportion of patients who were within therapeutic range for each group during this study, we binned tacrolimus levels every 5 days starting from the day of transplant. We collected records of held tacrolimus doses due to supratherapeutic levels. We also collected the number of tacrolimus dose was changes during the inpatient stay. Concerning the number of dose changes a patient experienced, we considered held doses as a change in the dose. Due to the imbalance in the numbers of patients with different genotypes, we normalized both total dose changes and total doses held by the number of patients with a specific genotype. We also assessed the percentage of total days of therapy each patient had to hold at least one dose of tacrolimus. In addition, dose changes for each patient during the inpatient stay were also assessed as normalized by each inpatient length of stay (LOS).

### 2.3 Genotyping

Genomic DNA was extracted from patients’ blood samples using DNAeasy Blood Mini kit (QIAGEN, Germantown, MD, United States) following the manufacturer’s instructions. RNase A was included in the purification to remove potential RNA contamination in DNA products. The genotypes of CYP3A5*3 (A- > G, rs776746), *ABCB1* exon 12 (C1246- > T, rs1128503), *ABCB1* exon 21 (G2677- > A/T, rs2032582), and *ABCB1* exon 26 (C3435- > T, rs1045642) were determined using polymerase chain reaction (PCR) - restriction fragment length polymorphism (RFLP) assays as previously described (Supplementary Table S1) (Fukuen et al., 2002; Owen et al., 2005; Dong et al., 2011). Patients were considered expressers of *CYP3A5* *1 as long as they were not homozygous for the *3 allele (*1/*1 or *1/*3). Genotyping of CYP3A4*1B (A- > G, rs2740574) was conducted on a QuantStudioTM 7 Flex system (Thermo Fisher Scientific, Wakthan, MA, United States) using a Taqman^®^ pre-validated genotyping kit, C__1837671_50 (Thermo Fisher Scientific) (Wang et al., 2011). The genotypes of CYP3A4*22 (C- > T, rs35599367) were determined using an allele-specific SYBR^®^ green real-time PCR assay as previously described (Papp et al., 2003). All genotyping experiments were conducted in duplicate.

### 2.4 Study endpoints

Pharmacokinetic endpoints were based on tacrolimus levels normalized to both the total daily dose of tacrolimus and the patient’s actual body weight. The goal range of serum tacrolimus levels for this study was defined as between 8 ng/mL and 12 ng/mL for the first 60 days after transplant as per our institution’s standard. After 60 days post-transplant, goal trough levels were determined by the preference of the treating physician. Analysis of time to therapeutic range was a simple comparison between the day number when the first therapeutic trough level was achieved as a continuous variable. Adverse event rates, including both acute kidney injury (AKI), tacrolimus-related neurotoxicity, and GVHD were collected. AKI was defined using KDIGO criteria (Khwaja, 2012). Neurotoxicity was defined by documentation of tremor or confusion attributed by the clinical team to be related to tacrolimus. GVHD was defined by documentation by the clinical team. For this study, only acute GVHD was included in our analysis. Patients without documentation of neurotoxicity or GVHD were assumed to not have experienced these adverse events.

### 2.5 Statistical analyses

All data analyses were conducted using R_3.3.2_ (The R Foundation; https://www.r-project.org). Patients’ characteristics were analyzed using descriptive statistics. Briefly, categorical data were presented as count and frequency; continuous data were summarized as mean and standard deviation (SD) except for age and The European Group for Blood and Marrow Transplantation (EBMT) risk score, which were summarized as median and range. Differences in categorical variables were analyzed using χ^2^ tests or Fisher’s exact tests. For continuous variables, Student’s t tests and one-way ANOVA were used to compare differences between two groups and among multiple groups where appropriate. For each SNP, the consistency between its distribution and the hyphenated Hardy-Weinberg principle was analyzed using χ^2^ tests. Potential statistical associations between different SNPs were also analyzed using χ^2^ tests using the R package genetics. Statistical tests were two-sided, and *p* values < 0.05 were considered statistically significant.

## 3 Results

### 3.1 Cohort demographics and genotype frequencies

In the present study, we assessed the impacts of various SNPs of *CYP3A4*, *CYP3A5*, and *ABCB1* on clinical outcomes of a cohort of 103 patients who were given oral tacrolimus for the prevention of GVHD after allo-HSCT. Full demographic features of these patients listed in Table 1. Median follow up was 98 days (interquartile range 92.5–100 days). In this cohort, tacrolimus levels were not associated with the use of voriconazole (*p* = 0.59) or steroids (*p* = 0.36).

**TABLE 1:** Demographic characteristics of patients included in this Study (N = 103).

Characteristics	Values
Age	
Median (Range)	55.6 (18.7–71.5)
Tacrolimus start day	
Day 2	88 (85.4%)
Day 3	15 (14.6%)
Day of first tacrolimus level	
Day 0	2 (1.9%)
Day 1	20 (19.4%)
Day 2	29 (28.2%)
Day 3	37 (35.9%)
Day 4	4 (3.9%)
Day 5	8 (7.8%)
Day 6	3 (2.9%)
Gender	
Female	39 (37.9%)
Male	64 (62.1%)
Ethnicity	
European ancestry	88 (85.4%)
Non-European ancestry	15 (14.6%)
Disease	
Acute Myeloid Leukemia	41 (39.8%)
Acute Lymphoblastic Leukemia	8 (7.7%)
Diffuse Large B-Cell Lymphoma	8 (7.7%)
Myelofibrosis	7 (6.8%)
Other	39 (38%)
Transplant Type	
Matched Sibling	35 (33.9%)
Matched Unrelated	54 (52.4%)
Mismatched Unrelated	1 (0.9%)
Umbilical Cord	13 (12.8%)
Cell Source	
Peripheral Blood Stem Cell Transplants	88 (85.4%)
Bone Marrow	2 (1.9%)
Umbilical Cord	13 (12.8%)
Preparative Regimen	
Myeloablative	13 (12.6%)
Reduced Intensity	90 (87.4%)
Comorbidity Index	3.1 (0–11)
Median (Range)	
ECOG Score	
0	46 (44.7%)
1	55 (53.4%)
2	2 (1.9%)
Karnofsky Score	
100	6 (5.9%)
90	38 (36.9%)
80	43 (41.7%)
70	14 (13.6%)
60	2 (1.9%)
EBMT	
Median (Range)	3.66 (1–6)

ECOG: eastern cooperative oncology group; EBMT: The European Group for Blood and Marrow Transplantation (EBMT) risk score.

Each SNP tested in this study was assessed for Hardy Weinberg Equilibrium. While the distribution of *CYP3A5* *3, *CYP3A4* *22, *ABCB1* exons 21 and 26 followed the Hardy Weinberg Equilibrium, *CYP3A4* *1B and *ABCB1* exon 12 deviated significantly (Table 2). In testing for linkage disequilibrium (Supplementary Table S2), an association was found between *CYP3A5* *1 and the *CYP3A4* *1B allele (*p* < 0.001; D’ = 0.48; *R* ^2^ = 0.18), *ABCB1* exon 12 and *ABCB1* exon 21 (*p* < 0.001; D’ = 0.66; *R* ^2^ = 0.22), *ABCB1* exon 12 and *ABCB1* exon 26 (*p* < 0.001; D’ = 0.45; *R* ^2^ = 0.20), and *ABCB1* exon 21 and *ABCB1* exon 26 (*p* < 0.001; D’ = 0.71; *R* ^2^ = 0.25). This is consistent with previous findings that *CYP3A4* *1B may be in linkage disequilibrium with *CYP3A5* *1 (Zeigler-Johnson et al., 2004; Miao et al., 2009). Additionally, three *ABCB1* SNPs were associated with each other in this cohort of patients (*ABCB1*: exon 12 and exon 21, *p* < 0.001; exon 12 and exon 26, *p* < 0.001; exon 21 and exon 26, *p* < 0.001), while *ABCB1* exon 21 was also associated with *CYP3A5* *1 and *CYP3A4* *1B (*p* = 0.001 and *p* = 0.045, respectively). We did not exclude variants from this study simply due to its deviation from Hardy Weinberg Equilibrium. Potential reasons for deviation from Hardy Weinberg Equilibrium include the relatively small sample size of this study, small variant allele frequency, and demographic homogeneity of this cohort.

**TABLE 2:** Genotypes of selected SNPs in *CYP3A5*, *CYP3A4*, and *ABCB1* in patients (N = 103).

SNP	Variant	Variant allele name	Copies of SNP detected			SNP allele frequency (%)	*p*-value*
			0	1	2		
rs776746	6986G>A	CYP3A5*3	1 (1.0%)	11 (10.7%)	91 (88.3%)	93.7	0.81
rs2740574	4713A>G	CYP3A4*1B	93 (90.3%)	7 (6.8%)	3 (2.9%)	6.3	<0.001
rs35599367	522–191 C>T	CYP3A4*22	94 (91.3%)	9 (8.7%)	0 (0%)	4.4	0.49
rs1128503	1236C>T	*ABCB1* Exon 12	45 (43.7%)	23 (22.3%)	35 (34.0%	45.1	<0.001
rs2032582	2677G>T/A	*ABCB1* Exon 21	51 (49.5%)	37 (35.9%)	15 (14.6%)	32.5	0.09
rs1045642	3435C>T	*ABCB1* Exon 26	31 (30.1%)	50 (48.5%)	22 (21.4%)	45.6	0.93

* *p*-value was from χ2 test based on the Hardy Weinberg Equilibrium (HWE) assumption. *p*-value <0.05 indicates that the genotype distribution in the study cohort significantly deviates from HWE., MAF, the minor allele frequency.

### 3.2 *CYP3A5* *1 expressers exhibited reduced normalized tacrolimus levels

**FIGURE 1:**
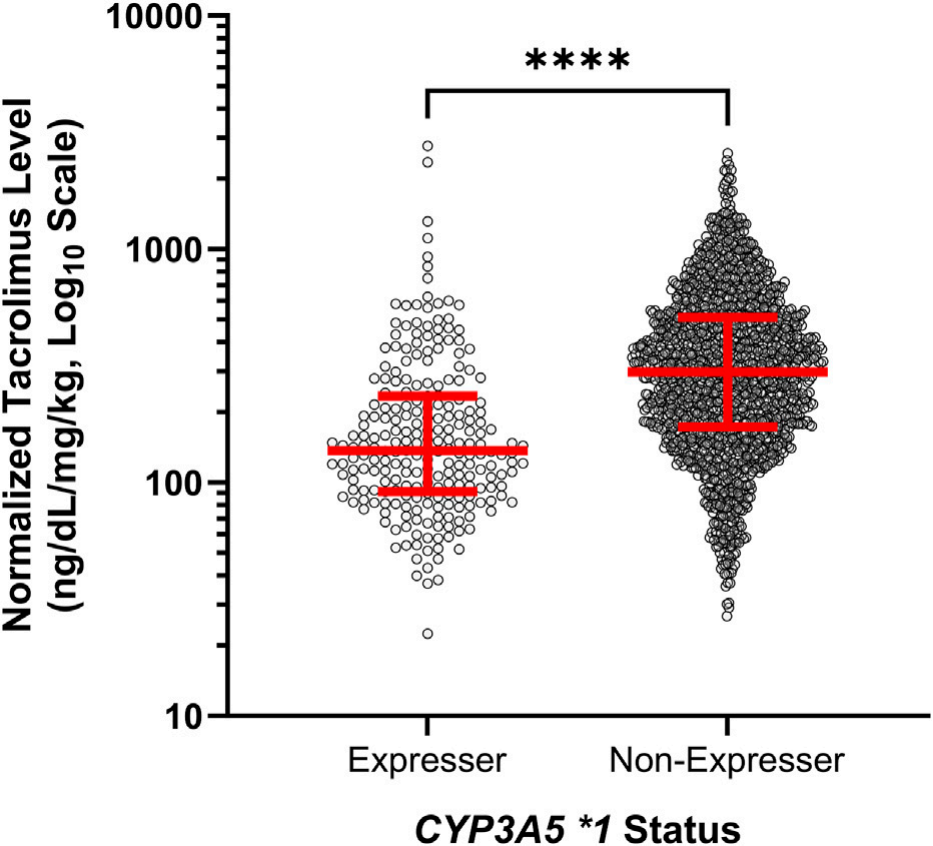
Expressers of *CYP3A5* *1 exhibited lower normalized tacrolimus concentration across the first 100 days of therapy. Red lines represent the median and interquartile range. ns, not significant; *, *p* < 0.05; **, *p* < 0.01; ***, *p* < 0.001, ****, *p* < 0.0001.

To assess the effect of our tested genomic variants with tacrolimus levels across the full study period, we calculated the median normalized tacrolimus level for each patient for the first 100 days of therapy. Univariate analyses demonstrated that median normalized tacrolimus level for each individual patient over the entire study period was statistically associated with each of *CYP3A4* *1B, *CYP3A4* *22, and *CYP3A5* *1 expression (*p* = 0.004, *p* = 0.03, *p* = 0.0009, respectively; Table 3). Multivariate analyses confirmed the association between normalized tacrolimus levels and *CYP3A5* *1 expression alone (Table 3; Figure 1). Based on these results, we focused further study on *CYP3A5* *1 expression.

**TABLE 3:** Correlation between alleles and tacrolimus concentrations.

Allele	Univariate			Multivariate			Variance explained
	Estimate	Standard error	*p*-value*	Estimate	Standard error	*p*-value*	
CYP3A5*1	−170.47	49.89	0.0009	−121.95	56.1	0.03	10.4%
CYP3A4*1B	−129.38	44.39	0.004	−70.04	49.14	0.16	7.8%
CYP3A4*22	143.41	65.67	0.03	116.86	62.92	0.07	4.5%
*ABCB1* Exon 12	−7.34	21.65	0.74				
*ABCB1* Exon 21	28.37	27.35	0.30				
*ABCB1* Exon 26	0.46	26.65	0.99				

* *p*-value was from linear regression on all normalized tacrolimus concentrations across the study period.

**FIGURE 2:**
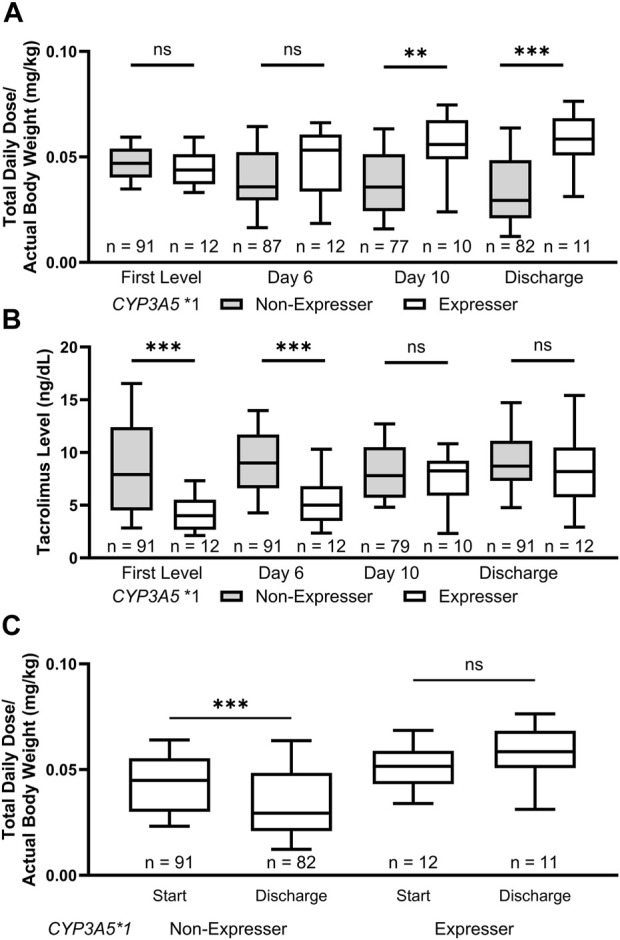
*CYP3A5* *1 expressers experienced lower initial tacrolimus levels and required increased doses of tacrolimus at discharge. **(A)** The weight adjusted tacrolimus dose increased during the inpatient stay for *CYP3A5* *1 expressers while the opposite was true for *CYP3A5* *3/*3 patients. **(B)** There was a significant difference in the tacrolimus levels achieved in patients grouped by *CYP3A5* genotype up until day 10 of therapy. **(C)** Patients with a *CYP3A5* *3/*3 genotype were discharged on lower weight adjusted doses of tacrolimus to their starting dose. ns, not significant; *, *p* < 0.05; **, *p* < 0.01; ***, *p* < 0.001, ****, *p* < 0.0001.

We then analyzed both weight normalized tacrolimus doses and tacrolimus levels at different time points during the inpatient stay of patients, including the first drawn level, day 6, day 10, and at discharge (±2 days). This analysis revealed a clinically important trend in dose requirements for *CYP3A5* *1 expressers. While the weight normalized tacrolimus dose at the time of the first level did not differ significantly based on *CYP3A5* genotype (*p* = 0.26; Figure 2A), the tacrolimus level at first assessment varied significantly (*p* < 0.001; Figure 2B). Over the course of the inpatient stay, *CYP3A5* *1 expressers received increasing tacrolimus doses compared to non-expressers (day 6: *p* = 0.17; day 10: *p* = 0.008; Discharge: *p* < 0.001) while the discrepancy in tacrolimus levels slowly diminished (day 6: *p* < 0.001; day 10: *p* = 0.32. Discharge: *p* = 0.37). *CYP3A5* genotype was clearly associated with changes in total daily dose of tacrolimus between the start of therapy and discharge, with the *CYP3A5* *3/*3 genotype associated with a decreased dose from drug initiation to discharge (*p* = 0.0002) (Figure 2C). The CPIC guidelines for *CYP3A5* genotype and tacrolimus dosing suggest a 1.5-2 times higher dose for patients who express *CYP3A5* *1 (Birdwell et al., 2015). At time of discharge, with statistically equivalent tacrolimus drug concentrations, patients who express *CYP3A5* *1 were stabilized on a median normalized tacrolimus dose 1.98 times higher than those who did not express *CYP3A5* *1.

**FIGURE 3:**
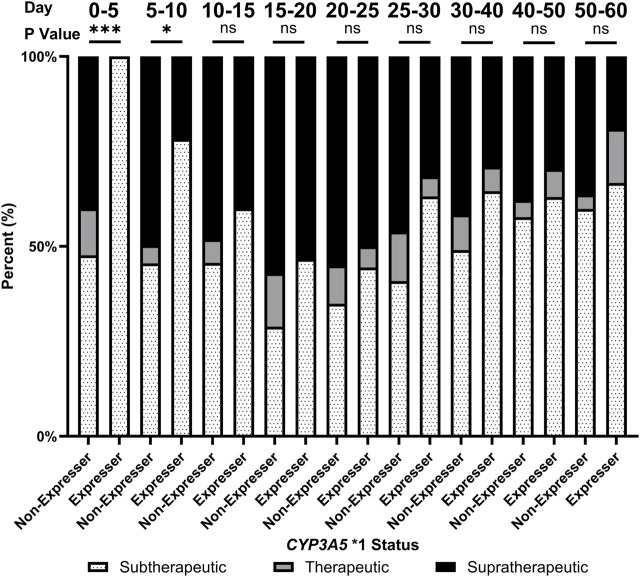
Proportion of patients achieving therapeutic levels of tacrolimus over the first 60 days of tacrolimus therapy by *CYP3A5* status. ns, not significant; *, *p* < 0.05; **, *p* < 0.01; ***, *p* < 0.001, ****, *p* < 0.0001.

In addition, *CYP3A5* *1 expressers spent significantly more time at subtherapeutic levels of tacrolimus compared to non-expressers throughout tacrolimus treatment (Figure 3). In the first 5 days after transplant, no *CYP3A5* *1 expressers (0/12, 0%) were at or above the therapeutic range. In comparison, 41.8% of *CYP3A5* *1 non-expressers were at or above the therapeutic range (38/91). Due to the significant impact of *CYP3A5* *1 expression on the pharmacokinetics of tacrolimus, expressers of *CYP3A5* *1 required more time to reach therapeutic range compared to non-expressers (13.9 ± 4.7 days vs. 9.9 ± 8.2 days; *p* = 0.003). Given this therapeutic complication, it is unsurprising that *CYP3A5* *1 expressers experienced longer inpatient stays (27.7 ± 24.1 vs. 20.0 ± 5.7 days, *p* = 0.02). *CYP3A5* *1 expressers also required fewer doses to be held (percentage of inpatient days held: 8.6% ± 12.6% vs. 30.8% ± 30.3%; *p* < 0.001) and, after normalization with the inpatient length of stay, required a lower number of dose changes during the inpatient stay compared to non-expressers (1.4 ± 0. 6 vs. 2.1 ± 0.8 changes/week; *p* = 0.008).

### 3.3 *CYP3A5* *1 expressers experienced similar, but delayed rates of AKI

To assess the effect of the tested genomic variants on clinical outcomes, we focused on the development of AKI and neurotoxicity in patients during the inpatient and outpatient stays. Expressers of *CYP3A5* *1 showed a clinical trend towards lower rates of AKI compared to non-expressers during the inpatient stay (7.7% vs. 25%; *p* = 0.06); however, rates of AKI over the entire period of this study does not differ by *CYP3A5* genotype (100% vs. 91.2%; *p* = 0.28). *CYP3A5* *1 expressers exhibited statistically similar rates of neurotoxicity (33.3% vs. 20.1%; *p* = 0.33) and GVHD (50.0% vs. 49.5%; *p* = 0.97) as non-expressers.

## 4 Discussion

Allo-HSCT is an integral therapy modality for the treatment of a number of hematologic malignancies requiring accurate and precise use of immune suppressing agents to prevent graft mediated harm to the patient. Multiple recent studies have confirmed the effect of *CYP3A5*, *CYP3A4*, and *ABCB1* in patients receiving tacrolimus via intravenous infusion in the setting of allo-HSCT (Hamadeh et al., 2019; Yoshikawa et al., 2021). However, very little data is available in the setting of oral tacrolimus. In this study, we prospectively enrolled 103 patients who received oral tacrolimus for GVHD prophylaxis following an allo-HSCT, as is standard of care at our institution.

Previous studies have demonstrated that *CYP3A5* *1 expressers and CYP3A4* 1B SNPs are associated with the pharmacokinetics and pharmacodynamic properties of tacrolimus (Semiz et al., 2011; Hamadeh et al., 2019; Zhu et al., 2020; Yoshikawa et al., 2021; Liu et al., 2022). Due to the potential linkage between the *CYP3A5* *1 expressers and CYP3A4* 1B SNP, it is difficult to fully characterize the effect of each individual allele (Sinues et al., 2007; Estrela et al., 2008). In our study, we determined that *CYP3A5* *1 expression was associated with an increased metabolism of tacrolimus leading to an increased dose requirement and time to reach therapeutic goals, while we were not able to identify an independent role for CYP3A4* 1B in tacrolimus metabolism (Musuamba et al., 2009; Liu et al., 2022). While most *CYP3A5* *1 expressers required doses increases following the first tacrolimus level to reach therapeutic goals, overall, these patients received less dose adjustments than those with the *CYP3A5* *3/*3 genotype. This observation could at least partially be attributed to clinicians’ reaction to high *versus* low tacrolimus levels. The clinician’s reaction to subtherapeutic tacrolimus levels, to an extent, dictates how much time is required to bring *CYP3A5* *1 expressers to therapeutic tacrolimus levels. Proactive genotyping of *CYP3A5* may allow clinicians to feel more confident in increasing the tacrolimus dose for carriers.

Genomic variations in *ABCB1* as well as the *CYP3A4* *22 SNP were not associated with tacrolimus pharmacokinetics. While *ABCB1* is not directly implicated in the metabolism of tacrolimus, it plays a vital role in the active transport of tacrolimus (Brunet et al., 2019; Hamadeh et al., 2019). Since our study included only patients who were initiated on oral tacrolimus, this gene was anticipated to be of great importance in tacrolimus absorption. However, there is no evidence from our study in support of the association between the selected three *ABCB1* SNPs and any clinically important endpoint as were found in previous studies (Hamadeh et al., 2019). We did observe a linkage association between SNPs in *ABCB1* exon 12 and both exon 21 and exon 26, which has been previously reported, therefore complicating our analysis (Allabi et al., 2005; Qiu et al., 2012). Further study is needed to better understand the clinical actionability of *ABCB1* in this setting.

AKI is one of the most common adverse events for patients prescribed tacrolimus and is associated with high drug concentrations. Here, *CY3A5* *1 expressers showed a clinical trend toward deceased rates of AKI during the inpatient stay, which dissipated over the course of the study. Expressers of *CYP3A5* *1 spend significantly more time at subtherapeutic tacrolimus levels during the inpatient stay; however, once these patients reached therapeutic goals, the difference in AKI rates among patients with different genotypes disappeared. This finding is of significance clinically as proactive genotyping may help to identify patients who are at higher risk of early AKI development (Seligson et al., 2023). Patients who are not expressers of *CY3A5* *1 may require closer renal monitoring during the inpatient stay while expressers of *CY3A5* *1 should be followed closely in the outpatient setting as they reach therapeutic goals.

Finally, it is important to note key limitations to this study. First, this was a single center study that, despite strong enrollment, resulted in a relatively small number of patients with variant SNPs. For example, our study cohort did not have many patients who expressed *CYP3A5* *1 or the *CYP3A4* *1B allele, which compromised our effort to separate the potential impacts of *CYP3A4* *1B. Our study also did not include SNPs in *CYP3A5*, notably *6 and *7, among other relevant SNPs in *CYP3A4* and *ABCB1* noted by the AMP and CPIC guidelines (Birdwell et al., 2015; Pratt et al., 2023). These SNPs were not well associated with tacrolimus metabolism when this study was designed. Further study should be sure to include these relevant SNPs in their analysis. From a clinical perspective, our study did not include any exclusions or measures of adherence to tacrolimus dosing after discharge. Data following discharge should then be considered as real-world data without a guarantee of adherence. Lastly, our study was reliant on chart review for many of the clinical variables assessed in this study, thus this study was not immune from errors in charting of the clinic course of a patient.

In conclusion, we provide evidence to support the role of *CYP3A5* *1 expression in the pharmacokinetics of oral tacrolimus in a large cohort of adult patients with allo-HSCT and reflect other recent findings in similar patient populations (Yamashita et al., 2016; Zhu et al., 2020). Patients expressing *CYP3A5* *1 required higher doses of tacrolimus to maintain therapeutic concentrations, suggesting that pharmacogenomics testing of *CYP3A5* may be used to assist in the dosing of oral tacrolimus in this clinical setting. Prospective studies in large cohorts of patients at multiple clinical sites are warranted to verify the findings presented here.

## Data Availability

The raw data supporting the conclusion of this article will be made available by the authors, without undue reservation.
