# Supplementary material for: CYP3A5 influences oral tacrolimus pharmacokinetics and timing of acute kidney injury following allogeneic hematopoietic stem cell transplantation
Source: Front Pharmacol. 2024 Jan 8;14:1334440. doi: 10.3389/fphar.2023.1334440 (PMC10800424; doi:10.3389/fphar.2023.1334440)
Supplement: Supplementary file 1 [file DataSheet1.docx]

Supplementary Material

CYP3A 4 and 5 Variants Influence Clinical Outcomes in Patients Receiving Oral Tacrolimus Following Allogeneic Hematopoietic Stem Cell Transplantation

Nathan D. Seligson^1,2,3^, Xunjie Zhang^1^, Mark C. Zemanek^1^, Jasmine A Johnson^1^, Zachary VanGundy^1^, Danxin Wang^4^, Mitch A. Phelps^3,5^, Julianna Roddy^1,2^, Craig C. Hofmeister^6^, Junan Li^3,7*^, and Ming J. Poi^1,2,3*^

*** Correspondence:** Dr. Junan Li, [li.225@osu.edu](mailto:li.225@osu.edu); Dr. Ming J. Poi, [poi.2@osu.edu](mailto:poi.2@osu.edu)

# Supplementary Data

None

# Supplementary Figures and Tables

For more information on Supplementary Material and for details on the different file types accepted, please see [here](https://www.frontiersin.org/guidelines/author-guidelines#supplementary-material).

## Supplementary Figures

None

## Supplementary Tables

**Supplementary Table 1.** Primers and probes used in genotyping

| **Gene** | **SNP ID** | **Primers/Probes** | **Assays** |
| --- | --- | --- | --- |
| *CYP3A4**1B  (A->G) | rs2740574 | Taqman^®^ assay C__1837671_50 | Taqman^®^ genotyping assay at 60°C |
| *CYP3A4**22  (C->T) | rs35599367 | wtF, 5’GTGTCTCCATCACACCCTGC3’  snpF, 5’GTGTCTCCATCACACCCCGT3’  CommonR, 5’GGTGTTATCAGGTGCCAGTG3’ | SYBR^®^ green real-time PCR assays at 60°C |
| *CYP3A5**3  (A->G) | rs776746 | Forward, 5’CTTTAAAGAGCTCTTTTGTCTCTC3’  Reverse, 5’CCAGGAAGCCAGACTTTGAT3’ | PCR with annealing at 60°C and extension at 72°C.  Digestion enzyme: Dde I |
| *ABCB1* exon 12  (C1246->T) | rs1128503 | Forward, 5’TATCCTGTGTCTGTGAATTGCC3’  Reverse,  5’CCTGACTCACCACACCAATG3’ | PCR with annealing at 60°C and extension at 72°C.  Digestion enzyme: Hae III |
| *ABCB1* exon 21  (G2677->A/T) | rs2032582 | Forward,  5’TGCAGGCTATAGGTTCCAGG3’  Reverse,  5’TTTAGTTTGACTCACCTTCCCG3’ | PCR with annealing at 60°C and extension at 72°C.  Digestion enzymes:  Kpn I, Ban I |
| *ABCB1* exon 26  (C3435->T) | rs1045642 | Forward,  5’TGTTTTCAGCTGCTTGATGG3’  Reverse,  5’AAGGCATGTATGTTGGCCTC3’ | PCR with annealing at 60°C and extension at 72°C.  Digestion enzyme: Dpn II |

**Supplementary Table 2.** Linkage between tested SNP genotypes.

| **SNP** | ***CYP3A5* *3** | ***CYP3A4* *1B** | ***CYP3A4* *22** | ***ABCB1* Exon 12** | ***ABCB1* Exon 21** |
| --- | --- | --- | --- | --- | --- |
| ***CYP3A4* *1B** | ***P* < 0.001**  (D’ = 0.48)  (R^2^ = 0.18) |  |  |  |  |
| ***CYP3A4* *22** | *P* = 0.25  (D’ = 0.99)  (R^2^ = 0.004) | *P* = 0.29  (D’ = 0.98)  (R^2^ = 0.003) |  |  |  |
| ***ABCB1* Exon 12** | *P* = 0.060  (D’ = 0.34)  (R^2^ = 0.01) | *P* = 0.36  (D’ = 0.18)  (R^2^ = 0.003) | *P* = 0.16  (D’ = 0.32)  (R^2^ = 0.006) |  |  |
| ***ABCB1* Exon 21** | *P* = 0.55  (D’ = 0.079)  (R^2^ = 0.001) | *P* = 0.86  (D’ = 0.025)  (R^2^ = 0.001) | *P* = 0.087  (D’ = 0.28)  (R^2^ = 0.01) | ***P* < 0.001**  (D’ = 0.66)  (R^2^ = 0.22) |  |
| ***ABCB1* Exon 26** | *P* = 0.28  (D’ = 0.20)  (R^2^ = 0.004) | *P* = 0.53  (D’ = 0.12)  (R^2^ = 0.001) | *P* = 0.078  (D’ = 0.39)  (R^2^ = 0.01) | ***P* < 0.001**  **(**D’ = 0.45)  (R^2^ = 0.20) | ***P* < 0.001**  (D’ = 0.71)  (R^2^ = 0.25) |

*P* values less than 0.05 indicate significant linkage disequilibrium.
